# Correction: Arsenic trioxide exposure accelerates colon preneoplasic aberrant crypt foci induction regionally through mitochondrial dysfunction

**DOI:** 10.1039/c8tx90004c

**Published:** 2018-02-23

**Authors:** Hichem Moulahoum, Belkacem Mohamed Amine Boumaza, Meriem Ferrat

**Affiliations:** a Laboratory of Cell and Molecular Biology , Faculty of Biological Sciences , University of Sciences and Technology Houari Boumediene (USTHB) , Algiers , Algeria . Email: hic_moul@hotmail.com; b Ege University , Faculty of Science , Biochemistry Department , 35100 Bornova, İzmir , Turkey

## Abstract

Correction for ‘Arsenic trioxide exposure accelerates colon preneoplasic aberrant crypt foci induction regionally through mitochondrial dysfunction’ by Hichem Moulahoum *et al.*, *Toxicol. Res.*, 2017, DOI: 10.1039/C7TX00213K.



## 

The corrected authorship list for this paper is shown above. Prof. Djerdjouri states that she was unaware of this submission and did not approve the manuscript.

In addition, the authors wish to add the following statement to this paper:

Histological analysis images for the control and DMH experiments in Fig. 2 were reproduced from the corresponding author's previous work; H. Moulahoum, A. L. Nagy, B. Djerdjouri *et al.*, *Inflammopharmacol*, 2017, ; https://doi.org/10.1007/s10787-017-0377-5, © Springer International Publishing AG 2017, with permission of Springer.

The authors note that the results reported in this *Toxicology Research* paper and the previous *Inflammopharmacology* paper were generated from some of the same animal study groups but analysis of the samples collected was performed separately.

The Royal Society of Chemistry apologises for these errors and any consequent inconvenience to authors and readers.

